# The correlation of intraoperative hypotension and postoperative cognitive impairment: a meta-analysis of randomized controlled trials

**DOI:** 10.1186/s12871-020-01097-5

**Published:** 2020-08-05

**Authors:** Xiaojin Feng, Jialing Hu, Fuzhou Hua, Jing Zhang, Lieliang Zhang, Guohai Xu

**Affiliations:** Department of Anesthesiology, The Second Affiliated Hospital of Nanchang University, Nanchang, 330006 China

**Keywords:** Intraoperative hypotension, Postoperative delirium, Postoperative cognitive dysfunction, Meta-analysis

## Abstract

**Background:**

There is no consensus on whether intraoperative hypotension is associated with postoperative cognitive impairment. Hence, we performed a meta-analysis to evaluate the correlation of intraoperative hypotension and the incidence of postoperative delirium (POD) or postoperative cognitive dysfunction (POCD).

**Methods:**

We searched PubMed, Embase, and Cochrane Library databases to find randomized controlled trials (RCTs) in which reported the relationship between intraoperative hypotension and POD or POCD. The retrieval time is up to January 2020, without language restrictions. Quality assessment of the eligible studies was conducted by two researchers independently with the Cochrane evaluation system.

**Results:**

We analyzed five eligible RCTs. Based on the relative mean arterial pressure (MAP), participants were divided into low-target and high-target groups. For the incidence of POD, there were two studies with 99 participants in the low-target group and 94 participants in the high-target pressure group. For the incidence of POCD, there were four studies involved 360 participants in the low-target group and 341 participants in the high-target group, with a study assessed both POD and POCD. No significant difference between the low-target and the high-target group was observed in the incidence of POD (RR = 3.30, 95% CI 0.80 to 13.54, *P* = 0.10), or POCD (RR = 1.26, 95% CI 0.76 to 2.08, *P* = 0.37). Furthermore, it also demonstrates that intraoperative hypotension prolonged the length of ICU stay, but did not increased the mortality, the length of hospital stay, and mechanical ventilation (MV) time.

**Conclusions:**

There is no significant correlation between intraoperative hypotension and the incidence of POD or POCD.

## Background

Postoperative cognitive impairment, including postoperative delirium (POD) and postoperative cognitive dysfunction (POCD), is a common neuropsychological disorder after surgery among patients [1]. Although neither POD nor POCD has a formal definition, it is recognized that they do exist [2]. POD is an acute change of patient’s attention, consciousness, perception or cognition, which occurs in several hours or days after the operation and its duration is usually short (a few days) [3, 4]; POCD is characterized by short-term disturbances in patients’ memory, executive functioning, personality or sleep, which usually appears in weeks or months after surgery and can last for months or even longer [5]. POD and POCD are leading to adverse results, including prolonged length of hospital stay, increased mortality and unexpected complications, which results in increased medical costs and decreased the quality of patient’s life [3, 6–8].

The underlying pathophysiology of POD or POCD is multifactor and complicated. Immutable risk factors, such as surgery types, age and baseline cognitive function have been identified [5, 7]. Although the definitive preventive or therapeutic measure of POD or POCD is still unknown, there are increasing studies shows that hypoperfusion of the brain caused by hypotension during the surgery may be one pathogenic mechanism [9–12].

Intraoperative hypotension, though lack of a widely accepted definition, often appears during anesthesia. It is usually manifested as mean arterial pressure (MAP) below the level of a predefined threshold during surgery [13, 14]. Hypotensive anesthesia brings a lot of obvious conveniences for some surgeries, including visualized anatomy, dry surgical area, and reduced blood loss during surgery [15]. Thus, intraoperative hypotension induced by anesthesia is also frequently observed. It seems plausible that the temporary brain perfusion of a patient becomes impaired when experiencing severe and prolonged low blood pressure, leading to cognitive impairment [16]. However, the specific correlation between intraoperative hypotension and postoperative cognitive function remains unclear and controversial. Evidence has shown a pivotal role for intraoperative hypotension in the development of cognitive impairment after surgery [9, 12, 17], whereas others have not [16, 18–23]. A single study cannot elucidate all factors while different study designs may cause selection bias.

Therefore, the goal of the current meta-analysis is to evaluate the association between intraoperative hypotension and the incidence of POD or POCD undergoing surgery.

## Methods

### Search strategy

We performed the meta-analysis following the recommendations of Preferred Reporting Items for Systematic Reviews and Meta-Analyses (PRISMA) guidelines [24]. A PRISMA checklist is available as a supplement (Table S1). Relevant studies were searched by the following databases: PubMed, Embase, and Cochrane Library databases. According to the predetermined strategy, for PubMed, the following terms were conducted with both MeSH and free terms: ((“Postoperative Cognitive Complications” OR “Postoperative Cognitive Dysfunctions” OR “POCD”) OR (“Delirium” OR “Postoperative delirium” OR “POD”)) AND (“Hypotension” OR “Low Blood Pressure”) AND (randomized controlled trial) in Title/Abstract. We also manually searched or any additional relevant studies to ensure that all related articles were included. The retrieval time is up to January 2020, and no language restrictions were applied.

### Study selection

The eligible criteria were as follows: (1) Randomized controlled trials (RCTs); (2) Participants who underwent surgical operations; (3) According to the relative MAP in the process of surgery, the patients were divided into the low-target and high-target groups; (4) The outcome was the occurrence of postoperative cognitive impairment (POD or POCD). The exclusion criteria were as follows: (1) Unavailable results for statistical analysis; (2) Reviews, meta-analysis, letters, etc.

### Data extraction and risk of bias

Two investigators (Feng and Hu) performed the processes of data extraction and the risk of bias independently, with a third investigator (Xu) to resolve the controversy. The following information was available after inclusion of eligible studies: first author, publication time, country, surgery/Anesthesia type, age of the subjects, MAP during surgery, duration of intervention, event numbers, methods and time of cognitive assessment, and outcomes. The following adverse events, including mortality, the length of hospital and ICU stay, and mechanical ventilation (MV) time were extracted as well. All data were collected using a standardized form. The risk of bias for the eligible study was conducted according to the Cochrane evaluation system [25]. This assessment included seven parts: random sequence generation, allocation concealment, blinding of participants and personnel, blinding of outcome assessment, incomplete outcome data, selective reporting, and other bias. Each project was classified as low risk, high risk, or unclear risk of bias.

### Outcome measures

According to the author’s definition, the primary outcomes were the incidence of POD and POCD. The secondary outcomes were the mortality, the length of hospital and ICU stay, and MV time.

### Statistical analysis

The results of the meta-analysis were analyzed by Review Manager 5.2. For dichotomous variables (POD or POCD incidence, mortality), we computed the relative risk (RR) with 95% confidence intervals (CI) by the Mantel-Haenszel method. For continuous data (length of hospital and ICU stay, and MV time), we used Inverse variance method to calculate Mean differences (MD) with 95% CI. In addition, we converted some continuous data, described as median and interquartile range (IQR) [21, 22], to mean and standard deviation (SD) by the formulas of Luo and Wan [26, 27]. Trial Sequential Analysis (TSA) allows the estimation of the required information size in a meta-analysis to detect or reject a certain intervention effect [28]. Thus, we used one-sided TSA to control random errors of primary outcomes by TSA v.0.9 beta software (http://www.ctu.dk/tsa/downloads.aspx), with a risk of 5% for type I error and a power of 80% were set.

Heterogeneity was evaluated with inconsistency (I^2^) statistic. Clinical heterogeneity relates to difference between studies in design factors (such as outcome definitions or blinding), while methodological heterogeneity originates from diversity in clinical factors (such as essential characteristics or surgical settings). Given a large amount of methodological and clinical heterogeneity, we selected a random-effect model in this study [29]. Subgroup analyses about the incidence of POCD were performed: (1) Cardiac surgery versus non-cardiac surgery; (2) General anesthesia versus epidural anesthesia. Significant statistical difference was defined as *P* value < 0.05.

### Assessment of publication bias and sensitivity analysis

We estimated the publication bias by a funnel plot with Egger’s tests when the number of included studies was more than ten [30]. Using the Peto odds ratio method, we conducted a sensitivity analysis of primary outcomes (the incidence of POD and POCD) to evaluate the stability of the results.

## Results

### Study characteristics

**Fig. 1:**
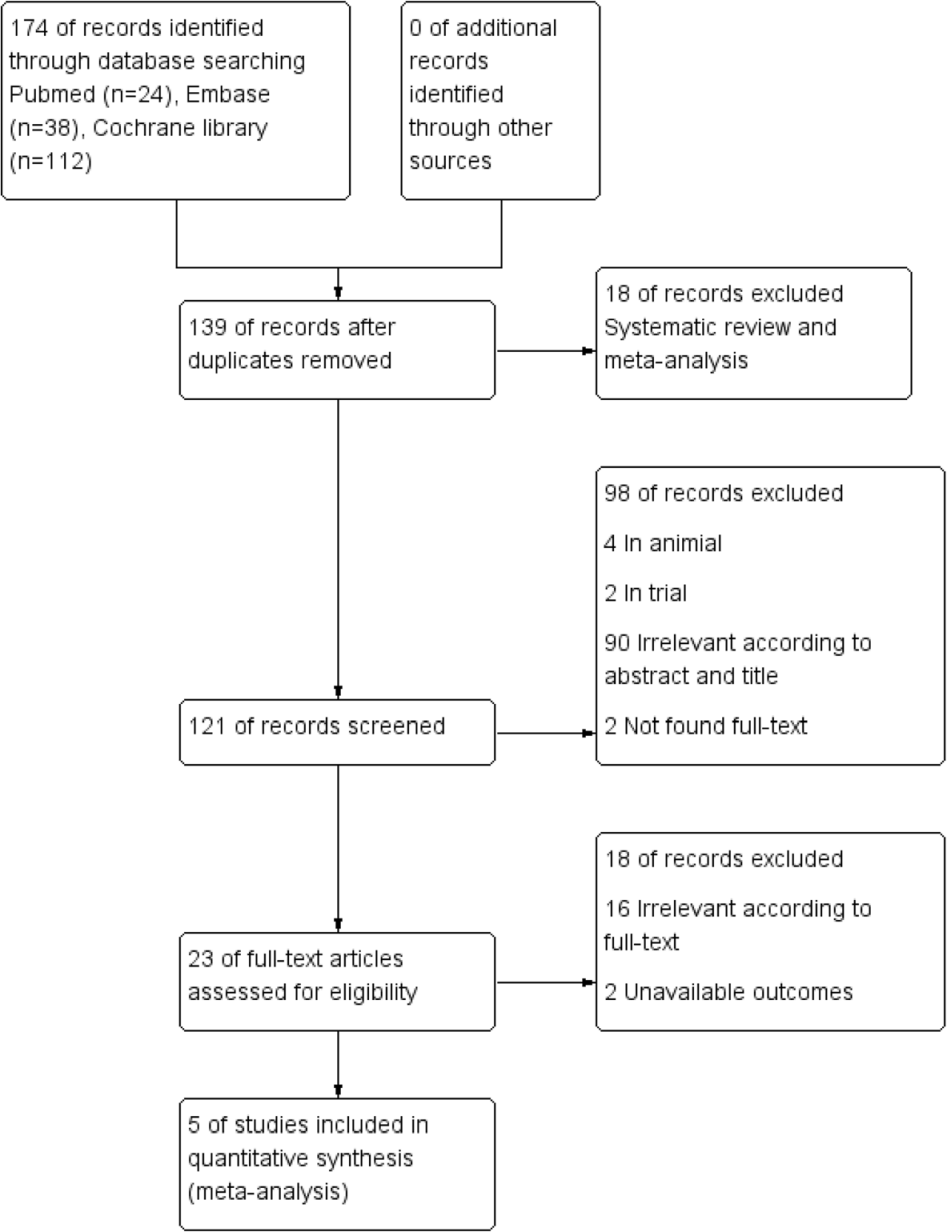
PRISMA diagram of study selection in this meta-analysis

Based on the above-mentioned search strategy, a total of 174 studies were identified (Pubmed = 24; Embase = 38; Cochrane Library = 112; Other = 0). Of these, 35 studies were removed due to duplication. According to the criteria mentioned above, the remaining 134 studies were excluded, and five studies were included in our final analysis [9, 20–23]. The flow diagram describing the study search is displayed in Fig. 1.

The main characteristics of included studies are summarized in Table 1. Two studies assessed the incidence of POD [9, 21], and four assessed POCD [20–23] (one assessed both POD and POCD [21]). Among them, three studies reported cardiac surgery [9, 21, 22], while the other two studies described non-cardiac surgery [20, 23]. The duration of intervention was about 1.5 to 4 h both in the low-target and high-target groups. Furthermore, the assessment methods of cognitive function were different, including neuropsychologic battery tests, Mini-mental state examination (MMSE) scores, International Study of Postoperative Cognitive Dysfunction (ISPOCD), and the Confusion Assessment Method adapted for the ICU (CAM-ICU) scale.

**Table 1:** Characteristics of the included studies

Author/ year	Country	Surgery type	Anesthesia type	Age	MAP during surgery (mm Hg)		Duration of intervention (min)		Low-target		High-target		Cognitive assessment		Outcome
					Low-target	High-target	Low-target	High-target	Events	Total	Events	Total	Method	Time (after surgery)	
Gold 1995 [20]	USA	CPB	General anesthesia	No reported	50–60	80–100	89.4 ± 31.5	84.9 ± 28.3	13	113	12	112	Neuropsychologic battery	6 months	POCD
Langer 2019 [21]	Italy	Non-cardiac surgery	General anesthesia	≥75	No-Target	≥90% of baseline	193 ± 117	198 ± 108	3	41	4	36	MMSE scores	3 months	POCD
									7	51	3	50	CAM-ICU scale	Once daily	POD
Siepe 2011 [9]	Germany	CPB	General anesthesia	≥55	60–70	80–90	101 ± 25	91 ± 30	6	48	0	44	MMSE scores	48 h	POD
Vedel 2018 [22]	Denmark	CPB	General anesthesia	≥18	40–50	70–80	94 ± 33	105.6 ± 77.4	8	89	5	75	ISPOCD battery	3 months	POCD
Williams 1999 [23]	USA	Total hip replacement	Epidural anesthesia	≥50	45–55	55–70	No reported	No reported	10	117	4	118	Neuropsychologic battery	4 months	POCD

Plus-minus values are mean ± SD

*Abbreviations*: *MAP* Mean arterial pressure, *CPB* Cardiopulmonary bypass, *MMSE* Mini-mental state examination, *CAM-ICU* Confusion Assessment Method Intensive Care Unit, *ISPOCD* International Study of Postoperative Cognitive Dysfunction, *POD* Postoperative delirium, *POCD* Postoperative cognitive dysfunction

### Risk of bias

**Fig. 2:**
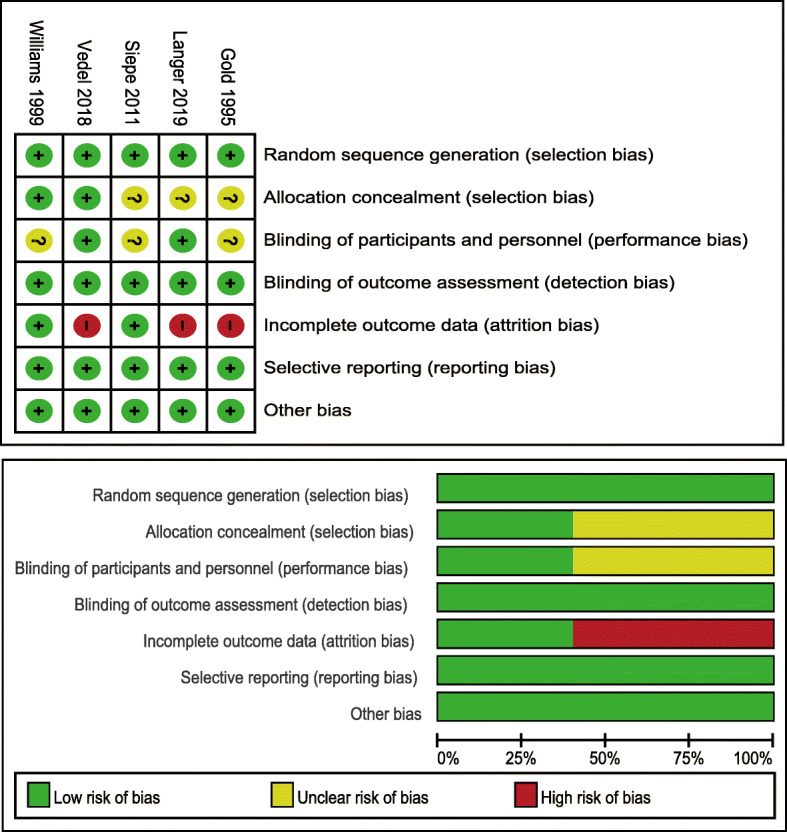
Risk of bias assessment for each study

The methodological bias of the eligible studies is presented in Fig. 2. Random sequence generation was considered as low risk of bias in all included studies, while allocation concealment was described in only two RCTs [9, 20, 21]. For performance bias, three RCTs reported an unclear risk [9, 20, 23], whereas the remaining two RCTs were assigned as low risk [21, 22]. All included studies were confirmed as a low risk of detection, reporting, and other biases. Three RCTs have a high risk of attrition bias [20–22]. Some participants of studies were possible to be lost due to the long-term follow-up period (up to 30 days).

### Primary outcome - incidence of POD

**Fig. 3:**
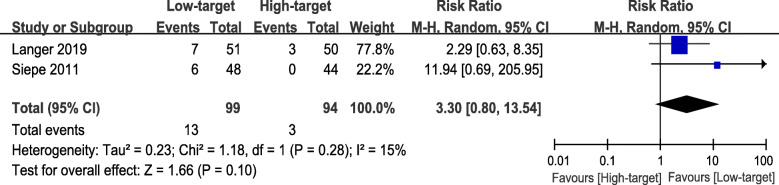
Forest plot of primary outcome - incidence of POD

There are two studies that reported the incidence of POD, and the data were described as the number of patients [9, 21]. Langer et al. [21] performed the assessment of POD in the late afternoon with the CAM-ICU scale, while Siepe et al. [9] conducted it on 48 h after surgery with MMSE scores. The study indicated a trend that patients in the low-target group (89 participants) had a higher incidence of POD than those in the high-target group (94 participants) (RR 3.30, 95% CI 0.80 to 13.54, *P* = 0.10, I^2^ = 15%), but the difference did not appear a clinical significance (Fig. 3). In TSA, the cumulative Z curve had crossed the traditional boundary line (Z = 1.96), but not crossed the TSA boundary line (Figure S1).

### Primary outcome - incidence of POCD

**Fig. 4:**
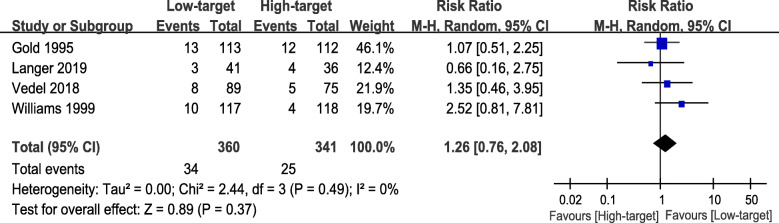
Forest plot of primary outcome - incidence of POCD

For the incidence of POCD, there were four studies included [20–23]. Three studies assessed POCD at over 3 months postoperatively [20, 21, 23], while one study reported the values of both 7 days and 3 months after surgery [22]. To avoid repeated counting and ensure the accuracy of the results, we only obtained the data reported 3 months postoperatively. In this meta-analysis, the incidence of POCD in the low-target group and the high-target group was 9.5 and 7.5%, respectively, showing no significant difference (RR 1.26, 95% CI 0.76 to 2.08, *P* = 0.37, I^2^ = 0%) (Fig. 4). In TSA, both traditional and TSA boundary lines (Z = 1.96) were not crossed; the estimated information size to reach the futility boundaries was 5064 randomized patients (Figure S1).

### Secondary outcomes

Four studies reported postoperative mortality of 638 patients [9, 20–22], in which no significant difference was observed between the low-target group and the high-target group (RR 0.86, 95% CI 0.14 to 5.37, *P* = 0.88, I^2^ = 44%). The length of hospital stay (described as days) data were available for 638 patients across four studies [9, 20–22]. It was noted that the value of the low-target group was lower than the high-target group, but the difference was so small that it did not have a statistical significance (MD 0.37, 95% CI − 0.17 to 0.91, *P* = 0.18, I^2^ = 0%). Data on the length of ICU stay (described as hours) was extracted from three studies that evaluated 537 patients [9, 20, 22], indicating that the time of the low-target group was longer than the high-target group (MD 1.82, 95% CI 0.83 to 2.82, *P* = 0.0003, I^2^ = 0%). Two studies reported MV time of the two groups [9, 22], and showed no significant difference (MD 0.40, 95% CI − 1.26 to 2.06, *P* = 0.64, I^2^ = 58%). The secondary outcomes of this study are shown in Table 2. Besides, we converted data described as median and IQR to mean and SD (Table S2).

**Table 2:** Secondary outcomes of this meta-analysis

Outcome	Number of studies	Number of participants		RR or MD	95% CI	Hterogeneity/I^2^	*P* value
		Low-target	High-target				
Mortality	4	322	316	0.86	0.14 to 5.37	44%	0.88
Length of hospital stay	4	322	316	0.37	−0.17 to 0.91	0%	0.18
Length of ICU stay	3	271	266	1.82	0.83 to 2.82	0%	0.0003
MV time	2	147	142	0.40	−1.26 to 2.06	58%	0.64

*Abbreviations*: *RR* Risk ratio, *MD* Mean difference, *CI* Confidence interval, *MV* Mechanical ventilation

### Subgroup analysis

**Fig. 5:**
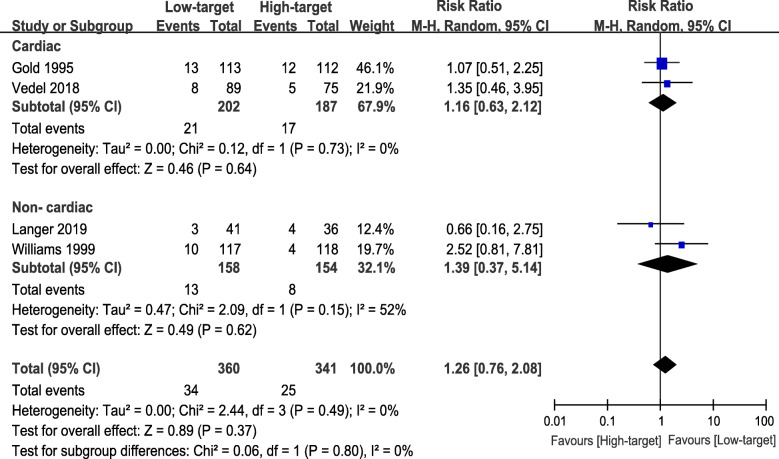
Subgroup analysis of cardiac surgery versus non-cardiac surgery

For POCD, there were two studies described cardiac surgery [20, 22] and non-cardiac surgery [21, 23], respectively. We further conducted a subgroup analysis of cardiac surgery versus non-cardiac surgery. When we excluded the results of non-cardiac surgery, no significant difference was found between the low-target and the high-target groups (RR 1.16, 95% CI 0.63 to 2.12, *P* = 0.64, I^2^ = 0%; 389 participants, Fig. 5). Also, there was no apparent difference between the subgroups (*P* = 0.80, Fig. 5). For POD, one RCT focused on cardiac surgery [9] and another addressed non-cardiac surgery [21]; thus, we did not compare the incidence.

**Fig. 6:**
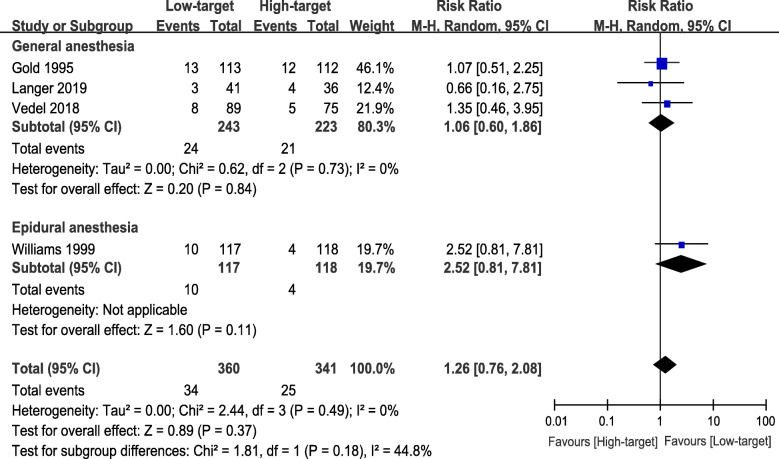
Subgroup analysis of general anesthesia versus epidural anesthesia

For POCD, three studies described general anesthesia [20–22], whereas one study described epidural anesthesia [23]. Further subgroup analysis on the POCD incidence of general and epidural anesthesia indicated no obvious significance between the low-target and the high-target group when epidural anesthesia was excluded (RR 1.06, 95% CI 0.61 to 1.86, *P* = 0.84, I^2^ = 0%; 466 participants, Fig. 6). No significant difference was observed in the subgroups (*P* = 0.18, Fig. 6). For POD, all patients of the included studies underwent general anesthesia [9, 21], so we did not perform a subgroup analysis.

### Assessment of publication bias and sensitivity analysis

Given that the number of the eligible studies was small, we did not assess publication bias [31]. Sensitivity analysis of the primary outcomes (the incidence of POD and POCD) by the Peto odds ratio method was yielded stably (POD: OR 3.67, 95% CI 0.86 to 15.62, *P* = 0.08 I^2^ = 12%; POCD: OR 1.30, 95% CI 0.75 to 2.25, *P* = 0.35, I^2^ = 0%).

## Discussion

In this study, we assessed the correlation of intraoperative hypotension and postoperative cognitive impairment following surgery and anesthesia. For the incidence of POD or POCD, the combined results illustrated no significant difference between the low-target and the high-target participants. TSA analyses showed that there was not enough information to confirm or reject the results, which requires a large number of randomized participants to achieve the boundary line. Furthermore, it demonstrated that intraoperative hypotension prolonged the length of ICU stay. Nevertheless, we did not notice obvious differences in the mortality, the length of hospital stay, and MV time between different groups.

Postoperative cognitive impairment (POD and POCD) is associated with high mortality and increased societal costs, which received increasing attention [1, 6–8]. Common concepts on the etiology are anesthesia-, surgery-, and patient-related factors [2, 3, 5]. Previous studies have reported that inflammation, neurotransmitter imbalance and sleep deprivation play an essential role in the pathogenesis of cognitive impairment [4, 6, 7, 32]. Moreover, some studies indicated that intraoperative hypotension was linked to the development of POD or POCD [9, 12, 17].

In this meta-analysis, we found that the mean ages of patients were more than 50 years old in most of the included researches. The possible explanation is that as population aging, more elderly patients are undergoing the operation, leading to a higher risk of cognitive impairment than younger patients. Furthermore, in this meta-analysis, two studies utilized the CAM-ICU scale [21] and MMSE scores [9] to assess the incidence of POD. The CAM-ICU scale had almost 100% sensitivity, specificity and interrater reliability [33], and MMSE scores had 96% sensitivity and 38% specificity [34]. For POCD, the incidence of three studies was elevated by neuropsychological tests [21–23], a sensitive method of evaluating the change and detecting beneficial results [35], while the remaining one used MMSE scores [9]. According to our study, the incidence of POD and POCD in the high-target group is only 3 and 7%, which were marginally lower than the reported rate in a systematic review (11–43% and 15–25%) [36]. Possible interpretations for this discrepancy include the considerable difference in test methods, the definition of POD or POCD, the baseline evaluation, and the control groups. Additionally, not only the occurrence of POD and POCD varied widely depending on the surgical variables, demographic as well as the clinical environment, but also increased with advancing age [3, 4].

Our meta-analysis suggested that intraoperative hypotension has no identified relationship on the incidence of POD, in line with previous studies on cardiac or non-cardiac surgery [9, 21]. However, two studies about colorectal [12] and surgical surgery [11] (a logistic regression and a retrospective cohort analysis) showed that intraoperative hypotension could significantly increase the incidence of POD. Possible reasons for the finding were that the definitions of hypotension used were different, and the above-mentioned two studies were not RCTs. Furthermore, a prospective cohort study on older patients during surgery found that both the degree and duration of intraoperative hypotension were not associated with POD, but fluctuations of intraoperative blood pressure was significantly related to the risk of POD [18]. Therefore, close monitoring and appropriate intervention of blood pressure during surgery seem to be crucial for preventing POD, which is to be clarified by RCTs with a larger sample size.

Our study concluded that there is no significant correlation between the POCD incidence and intraoperative hypotension. This conclusion is consistent with most studies [19–23, 37–39] except for a clinical, randomized study [9], which found that maintaining mean perfusion during cardiopulmonary bypass surgery at physiological values (80–90 mmHg) is associated with less early POCD. This discrepancy may be attributable to methodological issues concerning POCD: this study assessed it at 48 h after surgery, while others on over 3 months postoperatively; hence, Siepe et al. defined this cognitive impairment as early POCD. Additionally, regarding the effect of postoperative hypotension on postoperative cognitive impairment, no correlation was observed between postoperative hypotension and POCD [39], and no data was available about the relationship between postoperative hypotension and POD.

The result of our secondary outcomes indicated that intraoperative hypotension significantly prolonged the length of ICU stay, while not being followed by increased mortality, the length of hospital stay, and MV time. Furthermore, we also performed subgroup analysis for the effect of surgery type (cardiac versus non-cardiac surgery) and anesthesia type (general versus epidural anesthesia) on the incidence of POCD, which is consistent with a study revealing that the incidence is not associated with the type of anesthesia and surgery [40].

Several limitations of this study should be noted. First, the number of eligible studies was relatively small, resulting in a high risk of overestimation effects and a lack of publication bias assessment. Second, many factors such as surgery types, definitions of intraoperative hypotension or POD/POCD, intraoperative hypotension levels, and evaluation tools of POD or POCD varied among included studies. Thus, clinical heterogeneity was relatively high, which may weaken the reliability and precision of our conclusion. Third, given the fact that the incidence of POD and POCD were our primary outcomes, studies that did not contain POD or POCD data were excluded; thus, the application of our secondary outcomes may be limited. Therefore, the results of this meta-analysis should be further confirmed by much more high-quality studies.

## Conclusions

To our knowledge, this meta-analysis is the first systematic review to analyze the correlation of intraoperative hypotension and postoperative cognitive impairment, which provides a comprehensive summary of all currently available data on this crucial issue. Our study found that no significant relationship was seen between intraoperative hypotension and POD or POCD. Furthermore, it also demonstrated that intraoperative hypotension prolonged the length of ICU stay, but not increased the mortality, the length of hospital stay, and MV time. The current study has potential clinical implications for intraoperative blood pressure management, but other large, well-designed RCTs are needed to validate our conclusions in the future.

## Supplementary information

**Additional file 1: Table S1.** PRISMA 2009 Checklist.

**Additional file 2: Table S2.** Raw and converted data of the secondary outcomes.

**Additional file 3: Figure S1.** Trial sequential analysis of primary outcomes - incidence of POD (A) or POCD (B).

## Data Availability

All data generated or analyzed during this study are included in this published article.

## Abbreviations

CAM-ICU: Confusion Assessment Method adapted for the ICU
CI: Confidence intervals
ISPOCD: International Study of Postoperative Cognitive Dysfunction
IQR: Interquartile range
MAP: Mean arterial pressure
MD: Mean difference
MMSE: Mini-mental state examination
MV: Mechanical ventilation
OR: Odds ratio
POD: Postoperative delirium
POCD: Postoperative cognitive dysfunction
PRISMA: Preferred Reporting Items for Systematic Reviews and Meta-Analyses
RCTs: Randomized controlled trails
RR: Relative ratios
SD: Standard deviation
TSA: Trial sequential analysis
