# Supplementary material for: The correlation of intraoperative hypotension and postoperative cognitive impairment: a meta-analysis of randomized controlled trials
Source: BMC Anesthesiol. 2020 Aug 5;20:193. doi: 10.1186/s12871-020-01097-5 (PMC7409718; doi:10.1186/s12871-020-01097-5)
Supplement: Supplementary file 2 — Additional file 2: Table S2. Raw and converted data of the secondary outcomes. [file 12871_2020_1097_MOESM2_ESM.docx]

# Table S2 Raw and converted data of the secondary outcomes.

**Perioperative mortality**

| Author/year | Low-target | |  | High-target | |
| --- | --- | --- | --- | --- | --- |
|  | Events | Total |  | Events | Total |
| Gold 1995 | 5 | 124 |  | 2 | 124 |
| Langer 2019 | 1 | 51 |  | 1 | 50 |
| Siepe 2011 | 0 | 48 |  | 0 | 44 |
| Vedel 2018 | 0 | 99 |  | 4 | 98 |

**Length of hospital stay**

| Author/year | Low-target | | |  | High-target | | |
| --- | --- | --- | --- | --- | --- | --- | --- |
|  | Median (IQR) | Mean (SD) | N |  | Median (IQR) | Mean (SD) | N |
| Gold 1995 | / | 17 (25) d | 124 |  | / | 13 (14) d | 124 |
| Langer 2019 | 5 (3 - 7) d | 5 (3.1) d | 51 |  | 5 (2 - 8) d | 5 (4.6) d | 50 |
| Siepe 2011 | / | 12.2 (8.1) d | 48 |  | / | 12.6 (6.9) d | 44 |
| Vedel 2018 | 6 (5 - 8) d | 6.4 (2.3) d | 99 |  | 6 (5 - 7.5) d | 6 (1.9) d | 98 |

**Length of ICU stay**

| Author/year | Low-target | | |  | High-target | | |
| --- | --- | --- | --- | --- | --- | --- | --- |
|  | Median (IQR) | Mean (SD) | N |  | Median (IQR) | Mean (SD) | N |
| Gold 1995 | / | 77 (250) h | 124 |  | / | 60 ( 171) h | 124 |
| Siepe 2011 | / | 96 (93.6) h | 48 |  | / | 76.8 (57.6) h | 44 |
| Vedel 2018 | 21 (20 - 26) d | 22.4 (4.5) h | 99 |  | 21 (19 - 22) d | 20.6 (2.3) h | 98 |

**Mechanical ventilation time**

| Author/year | Low-target | | |  | High-target | | |
| --- | --- | --- | --- | --- | --- | --- | --- |
|  | Median (IQR) | Mean (SD) | N |  | Median (IQR) | Mean (SD) | N |
| Siepe 2011 | / | 11.9 (5.6) h | 48 |  | / | 10.3 (5.0) h | 44 |
| Vedel 2018 | 4.6 (2.9 - 6.7) h | 4.7 (2.9) h | 99 |  | 4.6 (2.3 - 7.9) h | 4.9 (2.3) h | 98 |

IQR: Interquartile range; SD: Standard deviation; N: Number of participants; d: Days; h: Hours.
