# Supplementary figures and images for: The correlation of intraoperative hypotension and postoperative cognitive impairment: a meta-analysis of randomized controlled trials
Source: BMC Anesthesiol. 2020 Aug 5;20:193. doi: 10.1186/s12871-020-01097-5 (PMC7409718; doi:10.1186/s12871-020-01097-5)

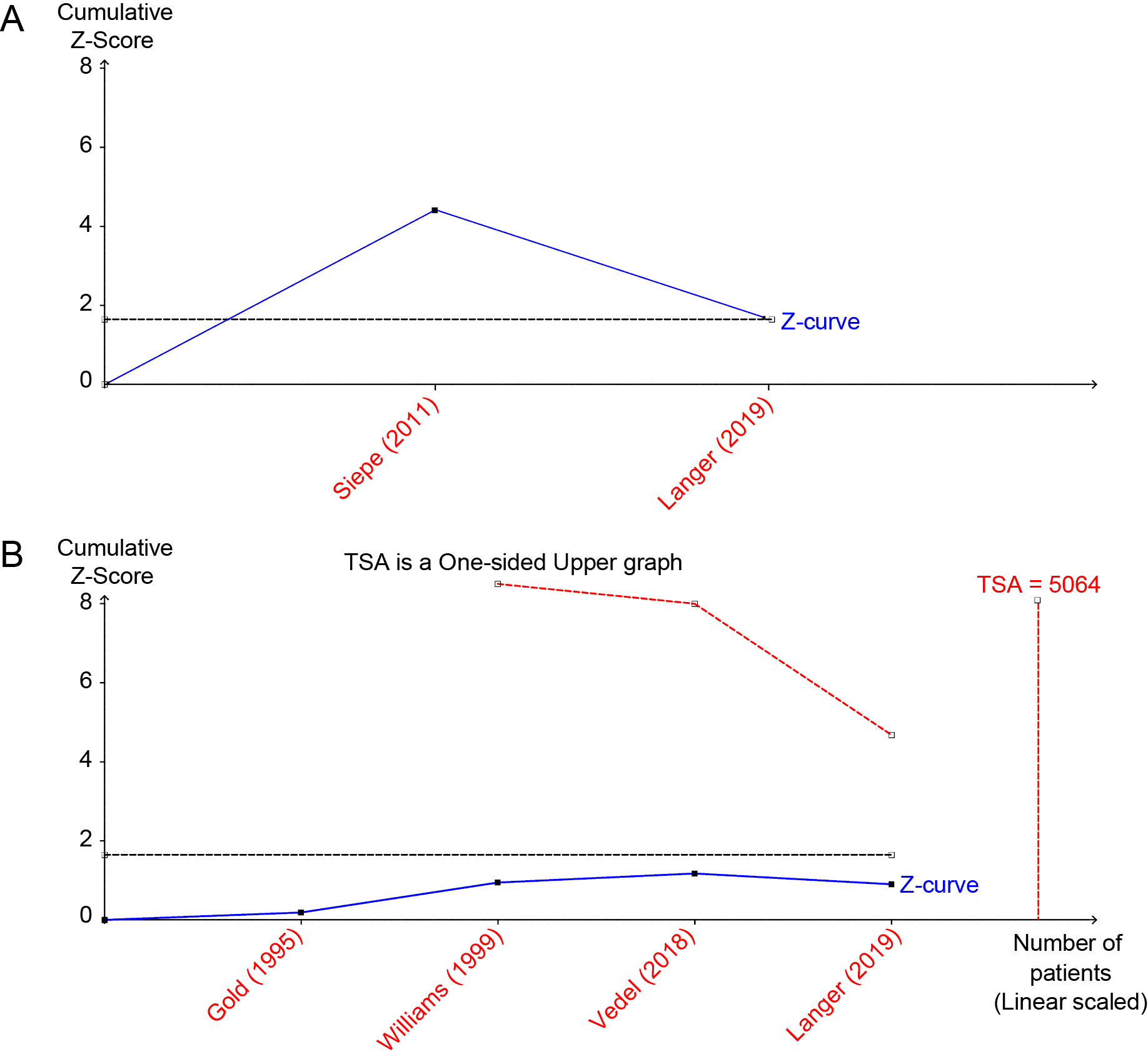


**Figure S1.** Trial sequential analysis of primary outcomes - incidence of POD (A) or POCD (B).

Supplement: Supplementary file 3 — Additional file 3: Figure S1. Trial sequential analysis of primary outcomes - incidence of POD (A) or POCD (B). [file 12871_2020_1097_MOESM3_ESM.docx]
